# Neural stem cells: ready for therapeutic applications?

**DOI:** 10.1186/2052-8426-2-31

**Published:** 2014-10-15

**Authors:** Simona Casarosa, Yuri Bozzi, Luciano Conti

**Affiliations:** Center for Integrative Biology, Università degli Studi di Trento, Via Sommarive 9, Povo-Trento, 38123 Italy

**Keywords:** Neural stem cells, Pluripotent stem cells, Cell therapy, Neuron, Neurodegenerative diseases, Neurodevelopmental disorders

## Abstract

Neural stem cells (NSCs) offer a unique and powerful tool for basic research and regenerative medicine. However, the challenges that scientists face in the comprehension of the biology and physiological function of these cells are still many. Deciphering NSCs fundamental biological aspects represents indeed a crucial step to control NSCs fate and functional integration following transplantation, and is essential for a safe and appropriate use of NSCs in injury/disease conditions. In this review, we focus on the biological properties of NSCs and discuss how these cells may be exploited to provide effective therapies for neurological disorders. We also review and discuss ongoing NSC-based clinical trials for these diseases.

## Review

### Introduction

Conventional pharmacological treatments for most neurodegenerative conditions relieve some symptoms but rarely vary the course of the disease or halt its progression. Grafting of human fetal tissue has provided a proof of concept for cell therapy approaches to neurodegenerative diseases in a number of clinical studies, including treatment of Parkinson’s and Huntington’s disease patients [1]. Nonetheless, this does not represent a practical route for large-scale therapeutic applications due to limited availability and quality of human fetal tissue, as well as for ethical considerations.

To this regard, in the last years, great media consideration has brought neural stem cell (NSC) research into the spotlight. Most of this attention has been raised by the stimulating prospects of NSCs application for cell replacement therapies for neurological disorders, engendering hopes and expectations in the public and, particularly, in patients. Despite the evident benefits pledged by the NSC field and some encouraging preliminary studies in animal models, there still remains a gap between theory and practice. Indeed, while stem cell-based therapies are the current standard of care for blood tumors and are gaining agreement in the treatment of epidermal and corneal disorders, applications for diseases affecting the nervous system yet represent a pioneering field, being in the early phases of clinical scrutiny. What is still missing to effectively translate NSC research into clinical applications? Although important scientific progresses in the field have been achieved, we still lack a profound understanding of the basic biology of NSCs and how to manipulate these cells to provide reliable, safe and effective outcomes in cell-replacement approaches.

**Figure 1:**
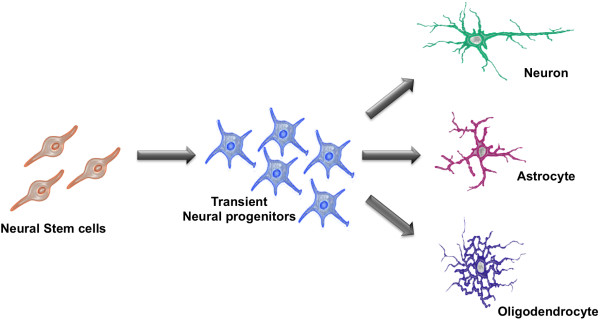
**Cardinal neural stem cell properties.**

**Figure 2:**
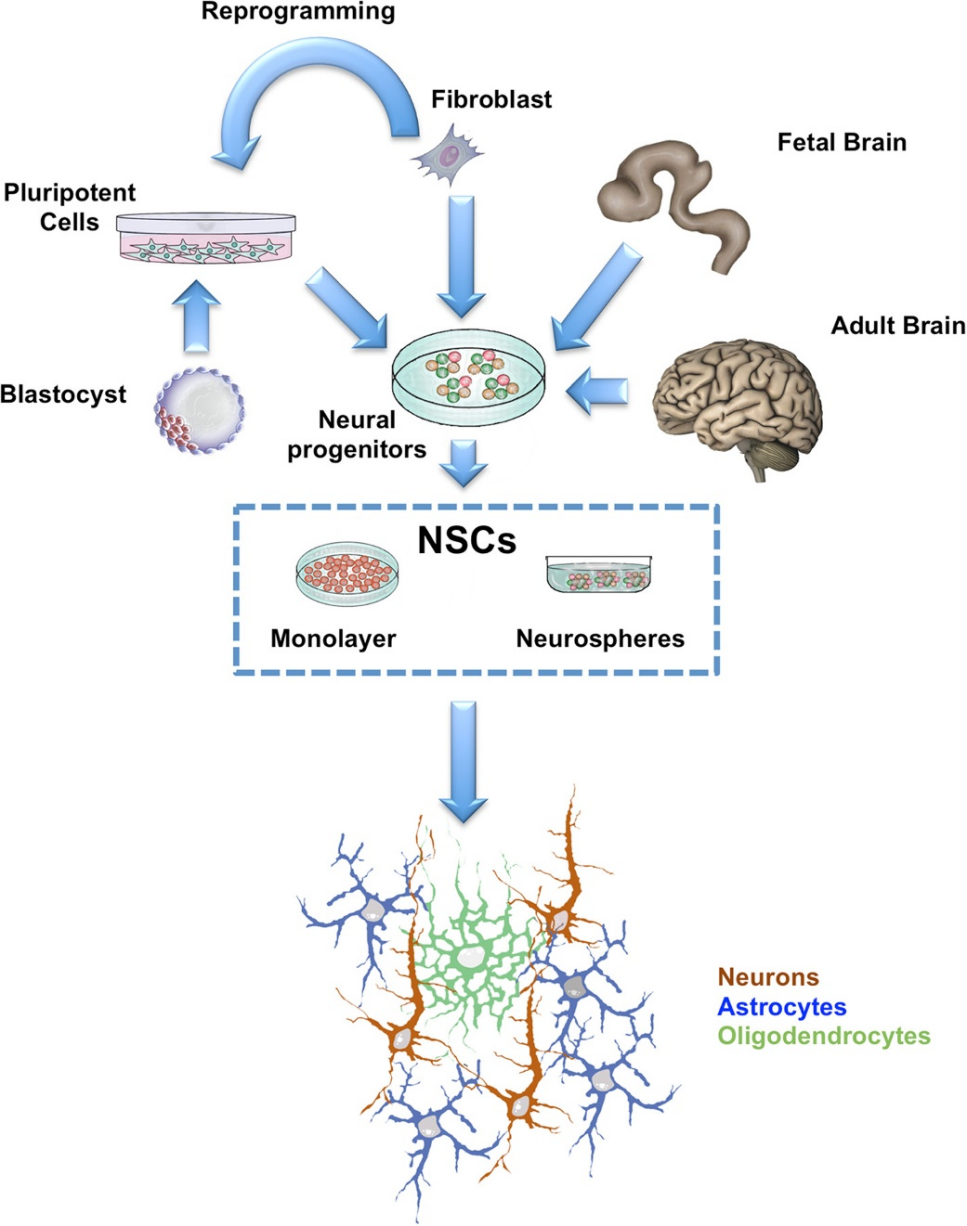
**Sources and** ***in vitro*** **growth protocols for neural stem cell generation and expansion.**

NSCs are immature cells present in the developing and adult Central Nervous System (CNS). Typically, NSCs are defined by three cardinal characteristics: self-renewal potential, neural tripotency (i.e., the capability to give rise to all of the major neural lineages: neurons, astrocytes and oligodendrocytes) and competence for *in vivo* regeneration (Figure 1; [2]). They have the potential to generate both neurons and glia of the developing brain and they also account for the limited regenerative potential in the adult brain. In the adult CNS, NSCs reside in defined regions (“neurogenic niches”) that sustain their multipotency and regulate the balance between symmetrical self-renewal and fate-committed asymmetric divisions [3].

Several studies have shown that NSCs can be extracted from neural tissue or generated from pluripotent cellular sources, genetically manipulated and differentiated *in vitro* [2]. In the past two decades, a variety of protocols for NSCs purification, generation and expansion in floating or adherent conditions have been described. However, beside these important progresses, the identification of the best sources for NSCs derivation and the optimization of approaches to stably proliferate them clonally *in vitro* still represents a major goal of NSC research.

Yet, for a realistic exploitation of NSCs for cell therapies, clinically-suitable NSC systems should hold specific key properties including (i) standardized production and scalability to good medical practice (GMP), (ii) karyotypic stability, (iii) ability to correctly integrate in the host tissue and (iv) differentiate into the required functional neural cells. In addition, NSCs should exhibit a reproducible, predictable and safe behavior following *in vivo* injection.

### NSCs in brain organogenesis and homeostasis

In the developing and adult CNS, different NSC populations dynamically appear following predetermined spatio-temporal developmental programs. Molecular and biological characteristics of NSCs greatly vary depending on the region and developmental stage considered [4].

Development of the vertebrate CNS starts with neural plate folding to originate the neural tube, consisting of radially elongated neuroepithelial cells (NEPs) [5]. NEPs develop definite identities and different fates depending on their positions along the rostrocaudal (R-C) and dorsoventral (D-V) axes of the neural tube. Patterning along the R-C axis leads to the initial distinction into prosencephalon, mesencephalon, rhombencephalon and spinal cord territories. NEPs are accountable for the first wave of neurogenesis in the neural tube. As development proceeds, NEPs convert themselves into another transitory NSC type, the so-called “radial glia” (RG) [6, 7]. This rapidly constitutes the main progenitor cell population in mid/late development and early postnatal life while disappearing at late postnatal and adult stages. Besides their ability to divide asymmetrically and to serve as progenitors of neurons and glia, RG cells constitute a scaffold on which neurons migrate in the developing brain. RG differentiation potential is less extensive compared to that of NEPs. Along with RG, another population of immature neural cells is constituted by Basal Progenitors (BPs) [8]. They are generated at early phases of development by NEPs and at later stages by RG. BPs mostly undergo one or two rounds of division, generating one or two pairs of neurons. Hence, BPs may be considered neurogenic transit-amplifying progenitors that specifically increase the production of neurons during restricted developmental time periods in definite brain areas (i.e. cerebral cortex).

At the end of neurogenesis (roughly at birth in mice), neurogenic RG cells are exhausted and residual RG cells are converted into a unique astrocyte-like subpopulation [9]. This population will make up the NSC pool of the adult brain, endorsed with neurogenesis and gliogenesis maintainance throughout adult life.

The concept that the adult brain retains the ability to self-renew some of its neurons has been broadly recognized in the last two decades and has represented a breakthrough in neurosciences. Pioneering studies from Altman and Das already reported the generation of new neurons in a variety of structures in the adult rat and cat including the olfactory bulb, hippocampus, and cerebral cortex [10]. However, their results were widely neglected until the early 1990s, when the formation of new neurons in adult rodent brain was clearly demonstrated [11, 12]. This led to the identification of the germinal zones of the adult brain. These are specialized niches located in the subventricular zone (SVZ) of the lateral ventricle wall and in the subgranular zone (SGZ) of the dentate gyrus of the hippocampus [3]. Whether NSCs reside in other regions of the adult mammalian brain is still disputed. Neuroblasts produced in the rodent SVZ migrate to the olfactory bulb following the rostral migratory stream (RMS), an anatomic structure well characterized in the rodent brain. The NSCs located in the SVZ, also called type B cells, generate actively dividing intermediate cells, named type C cells, which further divide giving rise to neuroblasts, referred to as type A cells that migrate away from the SVZ. These migrating neuroblasts are organized in chains that connect the SVZ to the olfactory bulb (constituting the RMS) where they gradually mature into functional GABAergic granule neurons. Fate-mapping studies actually reveal that type B cells are not developmentally restricted to neuronal lineages but can give rise also to glial progenies, suggesting they are authentic tripotent NSCs. The second germinal zone of the adult mammalian brain is the dentate gyrus of the hippocampus. Astrocyte-like NSCs, called type I progenitors, have been identified within the SGZ facing the dentate gyrus hilus. They share several properties with the type B cells of the adult SVZ, although they apparently exhibit a narrower developmental potential. Type I progenitors likely divide asymmetrically to produce immature proliferating progenitors, type II cells. These gradually differentiate into migrating neuroblasts that travel into the granule cell layer of the dentate gyrus, where they progressively mature into functional granule neurons. Differently from the type B cells of the SVZ, the progeny of type I progenitors does not migrate long distances, but remains localized in clusters closely connected to the parent cell. Additionally, hippocampal NSCs appear to be developmentally restricted to become granule neurons; currently, there is no evidence that type I progenitors can generate mature glial derivatives *in vivo*.

The discovery of NSCs and neurogenesis in the adult mammalian CNS has tremendously changed our view of the plasticity and function of the brain. This has prompted excitement for the possible exploitement of intrinsic neurogenic activity to cure brain diseases and rescue brain function after injury. Mobilization of endogenous NSCs has thus emerged as a potential therapeutic approach for neural repair. It is known that brain injury promotes the proliferation of adjacent NSCs, generating new astrocytes and neurons [13]. For example, focal ischemia transiently induces forebrain SVZ cell proliferation and neurogenesis. The NSCs in the SVZ and DG are also stimulated after traumatic brain injury or seizures [14], suggesting that adult neurogenesis may play a role in self-recovery mechanisms of the brain. However, the amount of spontaneously produced neuroblasts after brain injury is highly limited, and their survival and differentiation into mature neurons are far from obtaining regenerative effects.

It should be emphasized that, although our understanding of NSCs has increased dramatically over the past few years, there are still many major gaps regarding their *in vivo* control.

### NSCs for cell replacement approaches: requirements & available *in vitro*systems

A large number of studies have explored grafting behavior of several NSCs typologies (and their progeny) in a variety of preclinical studies and in some clinical investigations. Nevertheless, NSCs used for clinical applications should be safe, effective and accessible in large amount in GMP conditions. A variety of different sources for NSCs have been tested, including fetal- and adult CNS-derived NSCs, neural progenitors derived from pluripotent cells, and a range of non-neural stem cells, such as mesenchymal (MSCs) and bone marrow-derived (BMDSCs) stem cells. With these issues in mind, it should be remarked that up to now an ideal NSC system is not yet available to the clinic. Here, we will restrict our discussion to NSCs derived from neural tissue and from pluripotent stem cells. Advantages and disadvantages of each source and recent experimental evidence that highlight their potential use for clinical applications will be presented.

### Fetal- and adult-derived NSCs

The isolation of NSCs from their natural niches and their expansion in culture have been challenging issues, because the requirements to maintain these cells in their physiological state are yet poorly understood. In the early ‘90s, the identification of EGF and FGF-2 as key mitogens for NSCs led to set up culture conditions that support extended cell division of cells with NSCs properties [11, 12]. Since then, several studies reported that NSCs can be isolated from various regions of rodent (mouse and rat) and human brain at several developmental stages as well as from germinative areas of the adult brain. A widely used method is to culture NSCs as neurospheres (Figure 2; [15]). These are free-floating aggregates of neural progenitors, each, in theory, deriving from a single NSC. Their generation relies on neural tissue micro-dissection followed by exposure to defined mitogen-supplemented media. In such a procedure, primary cells are plated in low-attachment culture flasks in serum-free media supplemented with EGF and/or FGF-2. In these conditions, differentiating or differentiated cells are supposed to die, whereas NSCs respond to mitogens, divide and form floating aggregates (primary neurospheres) that can be dissociated and re-plated to generate secondary neurospheres. This procedure can be sequentially repeated several times to expand a NSC population.

Complementary to neurosphere culture is adherent culture, in which cells are more easily monitored and have better access to growth factors (Figure 2). In the last decade, several groups reported the generation and expansion of adherent NSC lines from neural tissue of rodent and human origin. According to this procedure, NSCs can be competently expanded as adherent clonal homogeneous NSC lines by exposure to specific mitogens, such as EGF and/or FGF2 [16, 17]. In these conditions, cells divide symmetrically, retaining their tripotential differentiation capacity. Adherent culture regimens have been shown to allow for cultures with less differentiated cells compared to the neurosphere assay, where cell–cell contacts and non-uniform mitogens exposure is thought to stimulate differentiation programs [2].

Although several studies have attempted to provide comparisons between fetal- and adult-derived NSCs, systematic side-by-side analyses are still few and do not allow to draw any solid conclusions. In fact, results might be hampered by culture conditions, especially for human NSCs. Nonetheless, major differences between fetal- and adult-derived human NSCs have been reported in terms of both biological and molecular properties. Fetal-derived human NSCs generally exhibit a shorter doubling time, a more extensive expansion potential *in vitro* and better integrative potential following grafting in animal models [18–21]. Noteworthy, substantial differences have been also described when comparing human NSCs derived from different brain areas of the same fetus [22].

### NSCs from pluripotent stem cells

Neuralization protocols applied to mouse and human pluripotent stem cells, including embryonic stem cells (ESCs) and induced pluripotent stem cells (iPSCs), allow for the generation of NSCs populations. ESCs are derived from the inner cell mass (ICM) of blastocyst stage mammalian embryos [23, 24]. They are characterized by an intrinsic capacity for self-renewal and the ability to generate all cell types derived from the three embryonic germ layers (pluripotency). In the last years, the advent of iPSC technology has completely revolutioned the “pluripotency” field, avoiding the requirement of embryos as source of rodent and, most importantly, of human pluripotent stem cells. Moreover, the use of iPSC opens new possibilities for studies of human development and disorders, further increasing the potential biomedical applications of this type of cells [25]. iPSCs are the product of a reprogramming procedure that allows the conversion of somatic cells directly into pluripotent cells [26, 27]. This technology is straightforward, robust and since its discovery has been implemented in terms of efficiency and reproducibility. iPSCs closely resemble ESCs with respect to expression of pluripotency markers, self-renewal potential, and multilineage differentiation potential. Both murine and human pluripotent stem cells can be exposed to neuralizing *in vitro* protocols to generate a large amount of NSCs or progenitor cells. In these conditions, pluripotent stem cells undergo progressive lineage restrictions similar to those observed during normal fetal development, leading to the generation of a range of distinct neural precursor populations [2].

Generally, two main procedures to generate NSCs from pluripotent stem cells have been developed. The first strategy relies on the formation of embryoid bodies (EBs), three-dimensional (3D) aggregates. EBs recapitulate many aspects of cell differentiation occurring during early mammalian embryogenesis and give rise to cells of the three germ layers, including neural cells. EBs dissociated and plated in adhesion on coated plastic surfaces in defined media will produce rosette-like neural cells corresponding to the NEPs of the developing brain [28, 29]. This NSC population can be subsequently enriched, although no efficient methods for their extensive expansion have been reported. EB-independent procedures based on adherent monolayer protocols have been also described [30, 31].

Electrophysiology studies have shown that pluripotent-derived NSCs efficiently generate fully mature neurons *in vitro*, as well as functionally integrated neurons after transplantation in the mammalian CNS [32, 33]. Nevertheless, major limitations to therapeutic applications of pluripotent-derived NSCs are represented by safety concerns and caveats about their clinical-grade production. Indeed, grafted pluripotent cells can form teratomas, implying that in a clinical setting residual undifferentiated pluripotent stem cells should be excluded from the cell preparation before grafting. Protocols for avoiding teratocarcinoma formation *in vivo* after transplantation of ESC/iPSCs-derived cells are under scrutiny. In this view, recent studies have reported the direct conversion of adult somatic cells into NSCs, thus opening a new path to generate NSCs without contamination of undifferentiated pluripotent stem cells [34].

The ability to generate patient-specific iPSCs and NSCs clearly provides enormous prospective for future personalized medicine, although too little is yet known about these cells to make any firm prediction.

### Possible therapeutic actions of grafted NSCs in different neurodegenerative conditions

Although the capacity of NSCs to divide and appropriately differentiate *in vitro* has attracted much attention for clinical translation, it does not assure that these cells functionally incorporate into the recipient tissue and produce efficient restoration of compromised functions after grafting. In order to generate therapeutic benefits in specific neurological diseases, grafted cells have to accomplish a certain grade of morphological, anatomical and functional integration into the impaired host CNS tissue.

Neurodegenerative disorders embody a heterogeneous collection of chronic and progressive diseases characterized by distinct aetiologies, anatomical impairments and symptoms [35]. Some of these disorders, such as Huntington’s disease (HD), are acquired in an entirely genetic manner. Alzheimer’s disease (AD), amyotrophic lateral sclerosis (ALS), and Parkinson’s disease (PD) mainly occur sporadically, although familiar forms caused by inheritance of gene mutations are known. On the other hand, the CNS can also be affected by other non-degenerative conditions, such as spinal cord injury and stroke, with no genetic heritable components.

By virtue of this extreme heterogeneity, different specific requirements should be envisaged when considering cell replacement as a possible therapeutic strategy. We can distinguish between (i) “neuronal” CNS degenerative disorders caused by a prominent loss of specific neuronal populations and (ii) “non neuronal” CNS degenerative conditions characterized by loss of non neuronal elements.

In the case of neuronal degeneration, the success of cell replacement strictly depends on the complexity and accuracy of the pattern of connectivity that needs to be restored. In PD, affected dopaminergic neurons in the substantia nigra (SN) exert a modulatory action on striatal target circuits mostly through the release of dopamine. This system is defined as “paracrine” and even a partial pattern repair may lead to a significant functional recovery in such conditions. Indeed, in PD the donor cells can be transplanted directly into the target region to circumvent the problem of long-distance neuritic growth in the adult CNS. Despite the ectopic location, if grafted cells are able to re-establish a regulated and efficient release of dopamine, they can lead to a clinically relevant functional recovery. However, cell-based treatment strategies are extremely difficult for other diseases such as HD, ALS, trauma, stroke, and AD, which are characterized by the need of a complex pattern repair.

Differently, “non neuronal” CNS degenerative syndromes such as multiple sclerosis (MS) characterized by severe inflammation, oligodendroglial degeneration and axonal demyelination, represent a good target for cell replacement, due to their limited requirements for pattern repair [36]. In MS, grafted cells should produce oligodendrocytes able to restore axonal myelination in order to lead to a functional rescue.

**Figure 3:**
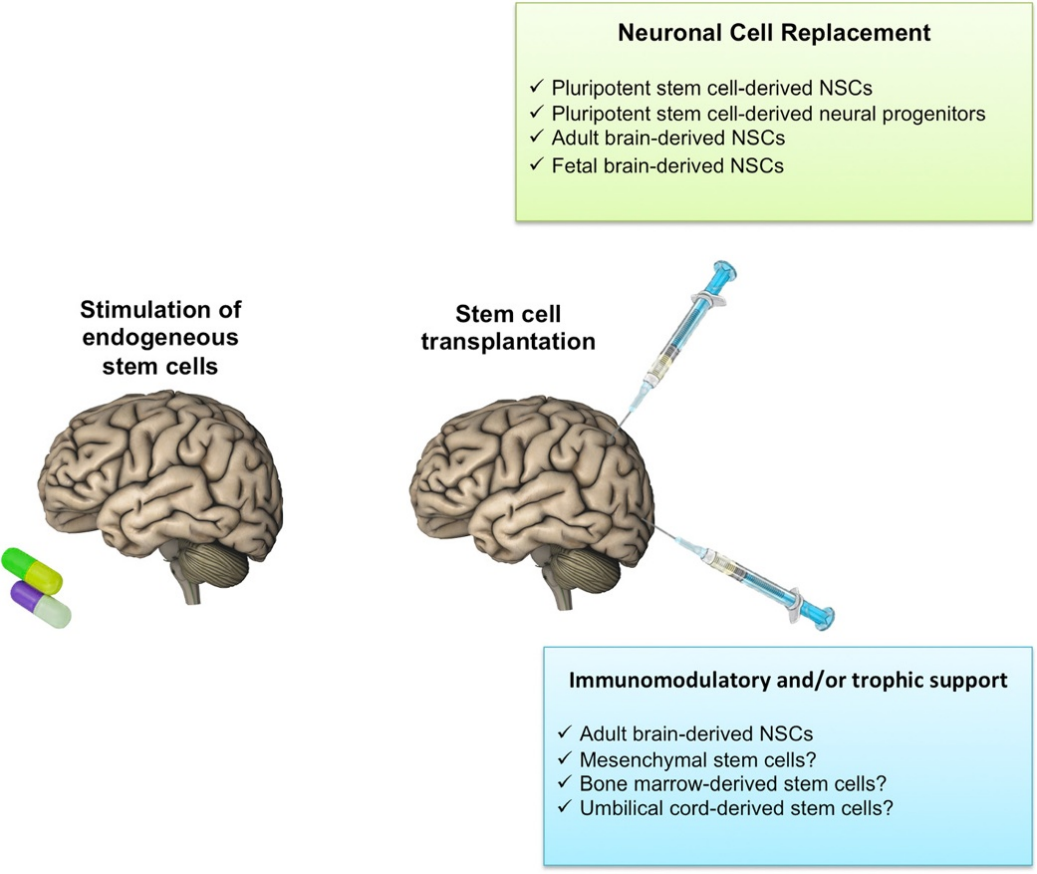
**Therapeutical strategies for neural stem cells exploitation in CNS diseases.**

It should also be emphasized that although pattern repair is critical to obtain permanent efficacy in the brain, cells transplanted into the brain may also be beneficial via the release of molecules that may either stimulate the regenerative potential of local cells (where present) or increase the survival of the remaining host elements, thus slowing disease progression (Figure 3; [37]). Also, immunomodulatory activity of the grafted cells could be of benefit in diseases such as MS where a prominent disease-associated inflammation contributes to the establishment and progression of the disease (Figure 3).

### Current clinical trials involving NSCs for neurodegenerative diseases

A large number of studies have explored the grafting behavior of several NSCs typologies (and their progenies) in preclinical studies. Moreover, a few attempts are already being made to translate these discoveries into the clinical setting. Currently, the registry and results database of publicly and privately supported clinical studies with human participants conducted around the world (http://www.clinicaltrial.gov/) reports 880 international clinical trials employing the use of stem cells for treatment of patients affected by several CNS disorders (query terms: “stem cells AND nervous system”) among which 89 are testing NSC injection approaches (query terms: “neural stem cell AND injection AND nervous system disease”). If restricting the search to non-tumor diseases, the database returns 51 studies (query terms: “neural stem cell AND injection AND nervous system disease NOT tumor”) with only 27 of these currently open. Interestingly, if we analyze these results more carefully, only 5 studies are actually open to explore the potential clinical relevance of NSCs while the remaining are testing the regenerative potential of mesenchymal stem cells whose actual uselfness in brain diseases is far from being a solid preclinical reality.

To date, few Phase I and II clinical studies employing NSCs (a dozen in highly debilitating CNS disorders; details reported in Table 1) have been performed, with the main objective to demonstrate safety and practicability and to explore the potential effectiveness of the treatments. These clinical trials are testing only few NSCs products, mainly consisting of fetal-tissue-derived allogenic NSCs. Among them, HuCNS-SC, a StemCells Inc. (Palo Alto, CA) product candidate, is a purified population of allogenic NSCs derived from human fetal (16–20 weeks) brain tissue, sorted using the CD133 marker and expanded in culture as neurospheres. These cells are routinely stored in a frozen state and reanimated before transplantation [38, 39]. In May 2006 a clinical trial (the first using human NSCs) was approved at Oregon Health and Science University (OHSU, Portland, OR, USA) for the use of these cells for lysosomal storage diseases (*ClinicalTrials.gov identifier,NCT00337636*), rare (one in 100,000 children) fatal autosomal recessive progressive neurological disorders caused by mutations in enzymes that ultimately lead to accumulation of neurotoxic lipofuscins. Patients generally lose their vision, develop seizures and dementia, and die before their teens. In this application NSCs serve as “Trojan Horses” to deliver enzymes to other cells within the brain, a concept established in rodent models of enzyme deficiencies. Preclinical studies indicated that intracerebral grafts into animal model of infantile neuronal ceroid lipofuscinosis (NCL, Batten’s disease) integrate into the host brain, produce and release the defective enzyme resulting in protection of affected neurons. In this open-label, dose-escalating phase I trial, single donor HuCNS-SC were injected into 6 patients with either infantile or late infantile NCL. Enrollment in the trial was limited to patients in the advanced stages of the disease (patients with significant neurological and cognitive impairment, whose developmental age was demonstrated to be less than two-thirds of their chronological age). Two dose levels were administered with the first 3 patients receiving a target dose of approximately 500 million cells and the other 3 patients receiving a target dose of approximately 1 billion cells. The cells were grafted directly into each patient’s brain (injections were performed into eight areas of each child’s brain) and patients received immunosuppression for 12 months after transplantation. The grafting procedure and combination with prolonged immune suppression were both well tolerated thus showing a favorable safety profile with transplanted cells, neurosurgical procedure, and immunosuppression regimen. In addition, transplanted cells showed long-term survival. Five patients (one died for disease) completed the 12-month Phase I study and were subsequently enrolled in a subsequent four-year, long-term observational study, with three of the five surviving to the end of the four-year study [40]. Three of the six patients transplanted with HuCNS-SC cells have now survived more than five years post-transplant, and it is noteworthy that each have stable quality-of-life measures, considering that they suffer a progressive neurodegenerative disorder. Assessment of the patients’ cognitive and neurological function revealed stable scores in some tests, but the clinical outcomes were generally consistent with the expected course of impairment associated with this neurodegenerative disease with no safety concerns attributed to the grafts. Examination of the brains from three patients who expired due to causes related to the underlying disease showed evidence of engraftment, migration and long-term survival of the HuCNS-SC cells following transplantation and the planned cessation of immunosuppression. Grafted cells provided widespread global replacement enzyme and bystander neuroprotection. Although these results look very promising, more detailed analyses and a longer patients’ follow up will be fundamental to draw more solid conclusions. In 2009, the Company completed the Phase I safety study and in October 2010 embarked on a Phase Ib safety and efficacy trial (*clinicaltrials.gov identifier no.NCT01238315*) in 6 children with less advanced Batten’s disease and therefore most likely to benefit from a timely neural stem cell transplant. However, the study was discontinued because of the failure to enroll patients meeting the study criteria (out of 22 initial prospects, none of them met the entry criteria).

**Table 1:** **Clinical trials involving NSCs for neurodegenerative diseases**

NCT number	Title	Recruitment	Conditions	Interventions	Sponsor/Collaborators	Phases	Enrollment	Start date	Completion date
**NCT00337636**	Study of HuCNS-SC Cells in Patients With Infantile or Late Infantile Neuronal Ceroid Lipofuscinosis (NCL)	Completed	Neuronal Ceroid Lipofuscinosis	Biological: HuCNS-SC	StemCells, Inc.	Phase 1	6	May 2006	September 2009
**NCT01005004**	Study of Human Central Nervous System (CNS) Stem Cells Transplantation in Pelizaeus-Merzbacher Disease (PMD) Subjects	Completed	Pelizaeus-Merzbacher Disease	Biological: HuCNS-SC cells implantation	StemCells, Inc.	Phase 1	4	November 2009	December 2012
**NCT01151124**	Pilot Investigation of Stem Cells in Stroke	Active, not recruiting	Stroke	Biological: CTX0E03 neural stem cells implantation	ReNeuron Limited	Phase 1	12	June 2010	March 2015
**NCT01217008**	Safety Study of GRNOPC1 in Spinal Cord Injury	Completed	Spinal Cord Injury	Biological: hES-derived GRNOPC1 implantation	Asterias Biotherapeutics, Inc.	Phase 1	5	October 2010	July 2013
**NCT01238315**	Safety and Efficacy Study of HuCNS-SC in Subjects With Neuronal Ceroid Lipofuscinosis	Withdrawn	Neuronal Ceroid Lipofuscinosis	Biological: HuCNS-SC cells implantation	StemCells, Inc.	Phase 1	0	November 2010	April 2011
**NCT01321333**	Study of Human Central Nervous System Stem Cells (HuCNS-SC) in Patients With Thoracic Spinal Cord Injury	Active, not recruiting	Thoracic Spinal Cord Injury|Spinal Cord Injury|Spinal Cord Injury Thoracic|Spinal Cord Trauma	Biological: HuCNS-SC cells implantation	StemCells, Inc.	Phase 1-2	12	March 2011	December 2015
**NCT01348451**	Human Spinal Cord Derived Neural Stem Cell Transplantation for the Treatment of Amyotrophic Lateral Sclerosis	Active, not recruiting	Amyotrophic Lateral Sclerosis	Biological: human neural spinal cord stem cells implantation	Neuralstem Inc.	Phase 1	18	January 2009	August 2014
**NCT01640067**	Human Neural Stem Cell Transplantation in Amyotrophic Lateral Sclerosis (ALS)	Recruiting	Amyotrophic Lateral Sclerosis	Biological: Human Neural Stem Cells implantation	Azienda Ospedaliera Santa Maria, Terni, Italy|Azienda Ospedaliero Universitaria Maggiore della Carita|Università di Padova Italy	Phase 1	18	December 2011	September 2016
**NCT01725880**	Long-Term Follow-Up of Transplanted Human Central Nervous System Stem Cells (HuCNS-SC) in Spinal Cord Trauma Subjects	Enrolling by invitation	Spinal Cord Injury	Observation	StemCells, Inc.	Phase 1-2	12	November 2012	March 2019
**NCT01730716**	Dose Escalation and Safety Study of Human Spinal Cord Derived Neural Stem Cell Transplantation for the Treatment of Amyotrophic Lateral Sclerosis	Enrolling by invitation	Amyotrophic Lateral Sclerosis	Biological: Human spinal cord stem cell implantation	Neuralstem Inc.	Phase 2	18	May 2013	April 2014
**NCT01772810**	Safety Study of Human Spinal Cord-derived Neural Stem Cell Transplantation for the Treatment of Chronic SCI	Not yet recruiting	Spinal Cord Injury (SCI)	Biological: Human spinal cord stem cells implantation	Neuralstem Inc.	Phase 1	8	May 2014	March 2016
**NCT02117635**	Pilot Investigation of Stem Cells in Stroke Phase II Efficacy	Not yet recruiting	Ischaemic Stroke|Cerebral Infarction|Hemiparesis|Arm Paralysis	Biological: CTX DP	ReNeuron Limited	Phase 2	41	June 2014	June 2017

A second open-label phase I clinical trial (*ClinicalTrials.govNCT01005004*) has been sponsored by StemCell Inc. at the University of California, San Francisco (UCSF) using HuCNS-SC brain transplantation for Pelizaeus-Merzbacher disease (PMD). PMD is a X-linked congenital leukodystrophy characterized by defective myelination [41]. Preclinical studies in animal models showed that intracerebral injection of HuCNS-SC produced oligodendrocytes leading to remylination of neurons affected by the mutated gene for PMD [42]. This phase I clinical trial has enrolled four young children with an early severe form of PMD. Each patient received a total brain dose of 300 million cells through two injections into the frontal white matter area of each hemisphere. Immunosuppression was administered for 9 months following transplantation. The patients have been followed for twelve months after transplantation; during this period they underwent regular neurological assessments and MRI analyses. Data regarding this clinical trial have been published in October 2012, indicating a good safety profile for the HuCNS-SC cells and the transplantation procedure [43]. Clinical assessment revealed small gains in motor and cognitive function in three of the four patients; the fourth patient remained clinically stable. Moreover, MRI inspections suggest myelination in the region of the transplantation, which progressed over time and persisted after the withdrawal of immunosuppression at nine months. Upon completion of the Phase I trial, all four patients have been enrolled into a 4-year long-term follow up study.

StemCells Inc. has also sponsored a Phase I/II clinical trial (*clinicaltrials.gov identifier no.NCT01321333*) at the University Hospital Balgrist (Zurich, Switzerland) for assaying safety and preliminary efficacy of intramedullary spinal cord transplantation of human HuCNS-SC neurospheres in subjects with thoracic (T2-T11) spinal cord trauma. The study began in March 2011 and includes 12 subjects who suffered spinal cord injury (SCI) in the 3 to 12 months prior to cell transplantation. Each subject received a total fixed dose of approximately 20 million cells injected directly into the thoracic spinal cord near the injury. The first patients have been transplanted with no safety concerns arising concerning the surgery and the cellular transplant [44]. This trial is estimated to end in March 2016. In November 2012 the consequential long-term follow up of the 12 patients subjected to HuCNS-SC transplantation has started and it will last until March 2018 (*clinicaltrials.gov identifier no.NCT01725880*).

Recently, also Neuralstem Inc. has received FDA approval to initiate a Phase I safety trial using proprietary NSCs for chronic spinal cord injury (*clinicaltrials.gov identifier no.NCT01772810*). The cell line used in this trial (NSI-566RSC) is derived from the cervical and upper thoracic regions of the spinal cord from a single 8-week human fetus after an elective abortion [45]. The cells are serially expanded as monolayer in serum-free media supplemented with FGF-2 as the sole mitogen to maintain proliferation and prevent differentiation. This trial will enroll up to eight SCI patients with thoracic lesions (T2-T12) one to two years post-injury with no motor or sensory function in the relevant segments at and below injury (complete paralysis). All patients in the trial will receive six injections close to injury site with the first four patients receiving 100,000 cells per injection and the second four patients 200,000 cells per injection. The patients will also receive immunosuppressive therapy, which will last for three months, as tolerated. Late this year, Neuralstem Inc. will also start an acute spinal cord injury trial in Seoul in collaboration with their South Korean partner CJ CheilJedang.

NSI-566 NSCs have also been used in clinical trials for the treatment of ALS (Lou Gehrig’s disease). In 2009 Neuralstem Inc. sponsored the first NSC-based phase I safety trial in ALS (*clinicaltrials.gov identifier no.NCT01348451*) at the Emory University School of Medicine (Atlanta, GA, USA). The trial enrolled 18 patients divided in 5 groups characterized by slightly different inclusion criteria and receiving different procedures in terms of site and number of injections (number of cells/injection: 100,000). The first group (cohort A) included patients with advanced ALS who are unable to walk, and received transplant injections in one or both sides of the lower spinal cord. The trial then progressed to patients who were still ambulatory. The first three of these patients (Cohort B) received five unilateral injections while the next three patients (Cohort C) received ten bilateral injections in the same lumbar region. In 2011 Neuralstem received approval to move into the cervical (upper back) stage of the trial. In this stage, two groups of three patients each were included. The first three of these patients (Cohort D) were ambulatory with some arm dysfunction and received grafts on one side of their neck. The last group (Cohort E) included ALS patients still ambulatory, who received both injections on one side of their neck and injections on both sides of their lower spine. The last patient in this Phase I trial was treated in August 2012 and the trial was concluded in February 2013. The clinical assessments on the first 12 grafted patients demonstrated no evidence of acceleration of disease progression with the planned 18 months post-transplantation follow up [46]. In April 2013, following conclusion of its Phase I FDA-approved trial, Neuralstem received approval to start a Phase II dose escalation and safety trial (*clinicaltrials.gov identifier no.NCT01730716*) to be performed in three centers: Emory University Hospital in Atlanta, Georgia, site of Phase I; ALS Clinic at the University of Michigan Health System, in Ann Arbor, Michigan, and Massachusetts General Hospital in Boston. This Phase II trial is designed to treat up to 15 ambulatory patients, in five different dosing cohorts, advancing up to a maximum of 40 injections, and 400,000 cells per injection based on safety. The first 12 patients will receive injections in the cervical region of the spinal cord only, where the stem cells could help preserve breathing function. The final three patients will receive both cervical and lumbar injections.

Another ALS Phase I clinical trial using allogenic fetal brain-derived human NSCs has been approved to the Azienda Ospedaliera Santa Maria (Terni, Italy) in June 2012. This study includes a total of 18 ALS patients that will be treated with intraspinal implanted allogeneic foetal-derived neurospheres (*clinicaltrials.gov identifier no.NCT01640067*).

Fetal NSCs are also being used for treatment of disabled ischemic stroke patients by the company ReNeuron Limited (UK). For this clinical application, ReNeuron uses its proprietary allogenic foetal-derived brain human NSCs (CTX0E03), which were derived from human fetal brain tissue following genetic modification with a conditional immortalizing gene, *c*-mycER [47]. This transgene generates a fusion protein that stimulates cell proliferation in the presence of a synthetic drug, 4-hydroxy-tamoxifen (4-OHT). The cell line is clonal, expands rapidly in culture and has a normal human karyotype (46 XY). In the absence of growth factors and 4-OHT, the cells undergo growth arrest and differentiate into neurons and astrocytes. The Phase I clinical trial (*clinicaltrials.gov identifier no.NCT01151124*) started on June 2012 at the Glasgow Southern General Hospital (Glasgow, Scotland). The study is designed to test the safety of CTX0E03 NSCs product by direct single dose injection into the damaged brains of 12 male patients 60 years of age or over who remain moderately to severely disabled 6 months to 5 years following an ischemic stroke. The trial will consider four ascending doses of CTX0E03 cells (4 dosage groups of three patients at each dose level receiving 2 million, 5 million, 10 million or 20 million cells). Clinical outcomes will be measured over 24 months and patients will be invited to participate in a long-term follow-up trial for a further 8 years.

To date, only one study has exploited the use of human ES-derived neural cells for the treatment of CNS injury in a clinical setting. In 2010, Geron Corporation started a Phase I Safety clinical trial with human ES cell-derived oligodendrocyte progenitors (GRNOPC1 cells) [48–50] in patients with neurologically complete, subacute spinal cord injury (*ClinicalTrials.gov identifier:NCT01217008*). The study was designed to test the safety of the Geron GRNOPC1 cell product by direct single dose (2 million cells) injection into the damaged spinal cord of five patients with neurologically complete spinal cord injuries (between 7 and 14 days post injury). The enthusiasm about this study was abated only a year later, when Geron suddenly and surprisingly stopped the trial and decided to give up on the stem cell division. In 2013 Asterias Biotherapeutics, Inc., a subsidiary of BioTime, purchased Geron’s stem cell division and announced that it will resume the spinal cord trial.

### Is there a rationale for stem cell-based treatments for neurodevelopmental disorders?

An increasing number of recent studies indicate that neurological and neuropsychiatric disorders including epilepsy, autism and schizophrenia may arise from altered brain development. According to this view, neurodevelopmental disorders may emerge from altered neurogenetic processes that lead to misplacement or loss of neurons and their connections in the postnatal brain. It is therefore not surprising that, in recent years, much attention has been paid to the possible use of NSCs to understand (and possibly treat) these pathologies. Currently, NSCs transplantation strategies have been essentially tested in rodent models of temporal lobe epilepsy (TLE). In both humans and rodents, TLE is accompanied by massive cell loss in the hippocampus and limbic areas [51]. In the past ten years, NSCs transplantation (both during the latent and chronic phases) has been widely tested as a tool to counteract cell loss and ameliorate TLE symptoms in rodents. NSCs are attractive as donor cells for grafting in TLE for a number of reasons: 1) they can be expanded in culture for extended periods; 2) they migrate extensively into the hippocampal layers; 3) they can differentiate into inhibitory GABAergic neurons as well as astrocytes secreting anticonvulsant factors (such as the glial cell-line derived neurotrophic factor GDNF); 4) they produce neurotrophic factors that can stimulate hippocampal neurogenesis from endogenous pools of NSCs [52]. Different strategies have been used to test the protective effects of transplanted NSCs, including transplantation of embryonic or adult hippocampal NSCs in both the KA and pilocarpine models of TLE [52]. Antiepileptic and neuroprotective effects have also been reported after transplantation of different types of stem cells. Grafting of human umbilical cord stem cells [53] or genetically-engineered bone marrow mesenchymal stem cells [54] have been shown to ameliorate seizures in the pilocarpine rat model of TLE.

Currently, research performed on animal models does not provide a rationale for the use of stem cells in neurodevelopmental disorders, with the only exception of epilepsy. Indeed, massive cell loss is detected in the epileptic brain, thus offering a rationale for cell replacement therapies. On the contrary, current research does not support the idea that stem cell transplantation may work in the case of other neurodevelopmental disorders such as autism, since no massive cell loss is observed in the brain of autistic patients. Accordingly, a Phase I trial to test the effect of stem cells transplantation in 20 patients with temporal lobe epilepsy is currently ongoing (*clinicaltrials.gov identifier no.NCT00916266*). The study, authorized on July 2008 to the Instituto do Cerebro de Brasilia (Brazil), has the aim to evaluate autologous bone marrow derived stem cells (BMDSCs) transplantation as a safe and potentially beneficial treatment for patients with temporal lobe refractory epilepsy. As primary outcomes were considered evaluation of seizure frequency, hippocampal volume and cognitive performance. Although the study should have been completed in June 2012, no results are currently available. Beyond the lack of solid results, it should be emphasized that the rationale for using BMDSCs transplantation in TLE patients is rather inappropriate, since these cells can not replace neural cell lost in these patients (see also below, “Clinical testing of non-neural cells for CNS diseases”).

More surprisingly, one completed and five ongoing trials have been approved to test the efficacy of stem cell transplantation in patients affected by autism spectrum disorders (query terms: “stem cells AND autism”) (details are reported in Table 2). None of these studies is actually injecting NSCs or pluripotent cells derivatives: they are focussed on using autologous BMSC (*clinicaltrials.gov identifier no.NCT01740869*, *NCT01974973*), adipose-derived MSC (*clinicaltrials.gov identifier no.NCT01502488*) and human cord blood mononuclear cells (*clinicaltrials.gov identifier no.NCT01343511*, *NCT01638819*, *NCT01836562*). Considering the young age and the overall number of patients enrolled (more than 350), the poor rationale and the total absence of published reports from these studies, great attention should be payed to these trials.

**Table 2:** **Clinical trials for neurodevelopmental disorderss**

NCT number	Title	Recruitment	Conditions	Interventions	Sponsor/Collaborators	Phases	Enrollment	Start date	Completion date
**NCT01343511**	Safety and Efficacy of Stem Cell Therapy in Patients With Autism	Completed	Autism	Biological: human cord blood mononuclear cells implantation|Biological: human cord blood mononuclear cells and human umbilical cord mesenchymal stem cells Injection	Shenzhen Beike Bio-Technology Co., Ltd.|Shandong Jiaotong Hospital|Association for the Handicapped Of Jinan	Phase 1-2	37	March 2009	May 2011
**NCT01502488**	Adipose Derived Stem Cell Therapy for Autism	Not yet recruiting	Autism	Procedure: Fat Harvesting and Stem Cell Injection	Ageless Regenerative Institute|Instituto de Medicina Regenerativa, S.A. de C.V.	Phase 1-2	10	October 2014	January 2017
**NCT01638819**	Autologous Cord Blood Stem Cells for Autism	Recruiting	Autism	Biological: Autologous Cord Blood Stem Cells Injection|Biological: Placebo	Sutter Health	Phase 2	30	August 2012	August 2014
**NCT01740869**	Autologous Bone Marrow Stem Cells for Children With Autism Spectrum Disorders	Recruiting	Autism|Autism Spectrum	Biological: Stem cells Injection	Hospital Universitario Dr. Jose E. Gonzalez	Phase 1-2	30	November 2012	Not specified
**NCT01836562**	A Clinical Trial to Study the Safety and Efficacy of Bone Marrow Derived Autologous Cells for the Treatment of Autism	Recruiting	Autism	Biological: Autologous Cord Blood Stem Cells Injection	Chaitanya Hospital, Pune	Phase 1-2	100	March 2011	April 2014
**NCT01974973**	Stem Cell Therapy in Autism Spectrum Disorders	Recruiting	Autism Spectrum Disorders	Procedure: Autologous bone marrow mononuclear cell transplantation|Procedure: Autologous bone marrow mononuclear cell transplantation in patients with autism	Neurogen Brain and Spine Institute	Phase 1	150	August 2009	October 2014

### Retinal dystrophies and stem cell treatments

Another field in which a strong interest has been raised for cell treatments based on NSCs or cell derivatives of human pluripotent stem cells is represented by retinopathies. Many studies are undergoing in order to characterize the appropriate source of cells for these therapeutic approaches. The retina is subject to different degenerative diseases, which can be both age related (such as AMD, Age-related Macular Degeneration) and inherited (LCA, Leber’s Congenital Amaurosis, and RP, Retinitis pigmentosa). These are all characterized by degeneration of photoreceptors and/or retinal pigmented epithelium (RPE cells). A number of different therapeutical approaches have been meaningfully explored to cure human photoreceptor degenerations. These include environmental modifications (nutrient supplementation and avoidance of light), drugs (i.e. neurotrophic factors), gene therapy to provide the healthy version of the mutated gene, retinal prostheses [55].

A large body of transplantation studies has been carried out in order to assess the molecular features characterizing cells with the ability to integrate and generate functional photoreceptors in degenerating retinae. These studies defined that, in rodents, integration of donor cells is best obtained by using post-mitotic photoreceptor precursors [56, 57]. They also showed that, for donor cells to integrate in retinal tissue, they should have specific molecular characteristics and should be derived from retinae that have not reached complete maturation. Despite the promise, the low numbers of integrating cells impaired a real functional recovery in the transplanted eyes, even if some restoration of vision was observed [58]. Moreover, a possible transition to the clinic would be blocked by the obvious ethical concerns in the use of human retinal progenitor cells. For these reasons, many reseachers turned their attention to obtaining postmitotic photoreceptor precursors *in vitro*, by differentiation of pluripotent cells such as ESCs and iPSCs. Integration capabilities of the *in vitro* differentiated cells have also been tested by subretinal injections in mice [59–61]. All these studies assessed terminal differentiation and integration of pluripotent cells-derived photoreceptors and, when possible, functionality, although showing alternative results.

To date, the clinicaltrials.gov registry reports 50 international clinical trials employing different stem cells types (query terms: “stem cells AND retinopathy”), mostly autologous BMSCs, for treatment of patients affected by retinal dystrophies. Among these, an age related macular degneration (AMD) Phase I/II trial sponsored by Stem Cell Inc. is ongoing in three US centers: (Byers Eye Institute at Stanford, Stanford Hospital and Clinics, Palo Alto, California; New York Eye and Ear Infirmary, New York; Retina Foundation of the Southwest Recruiting Dallas, Texas) (*clinicaltrials.gov identifier no.NCT01632527*). This study, involving 16 patients affected by geographic atrophy secondary to AMD, is designed to investigate the safety and preliminary efficacy of unilateral subretinal transplantation of HuCNS-SC cells by means of a single transplant procedure. Immunosuppressive agents will be administered orally to all subjects for a period of three months after surgery. Participants will be monitored for complications as well as structural evidence of successful engraftment and changes to vision.

Interestingly, the field of retinopathies represents the arena where the largest number of human ESC-derived products is being tested in the clinics. Currently, seven trials are open employing terminally differentiated human ESC-derived hRPE (details are reported in Table 3). Six of these studies are sponsored by Advanced Cell Technology (ACT; USA) and CHA Bio & Diostech (South Korea) for the use of the ACT’s MA09-hRPE cells for treatment of Stargardt’s Macular Dystrophy patients (SMD; *clinicaltrials.gov identifier no.NCT01469832*, *NCT01345006* and *NCT01625559*), dry AMD patients (*clinicaltrials.gov identifier no.NCT01674829*, *NCT01344993*) or Myopic Macular Degeneration patients (MMD; *clinicaltrials.gov identifier no.NCT02122159*). In these studies, the number of grafted cells varies between 50,000 and 200,000 cells in order to define the optimal dosage. The preliminary results of two of these trials have been published, showing safety and some promising efficacy in vision restoration. For this reason, MA09-hRPE cells from ACT have been recently granted by the FDA the orphan drug designation for use in the treatment of Stargardt’s Macular Dystrophy (SMD). This represents the first time that orphan drug status has been granted for the use of an embryonic stem cell derived therapy in treating an unmet medical need. As a result, ACT is entitled to obtain several benefits aimed at forstering clinical exploitation of this cell product, including tax credits, access to grant funding for clinical trials, accelerated FDA approval and allowance for marketing exclusivity after drug approval for a period of as long as seven years.

**Table 3:** **Clinical trials involving stem cells for retinal dystrophies**

NCT number	Title	Recruitment	Conditions	Interventions	Sponsor/Collaborators	Phases	Enrollment	Start date	Completion date
**NCT01344993**	Safety and Tolerability of Sub-retinal Transplantation of hESC Derived RPE (MA09-hRPE) Cells in Patients With Advanced Dry Age Related Macular Degeneration	Recruiting	Dry Age Related Macular Degeneration	Biological: MA09-hRPE implantation	Advanced Cell Technology	Phase 1-2	16	April 2011	December 2014
**NCT01345006**	Sub-retinal Transplantation of hESC Derived RPE (MA09-hRPE) Cells in Patients With Stargardt’s Macular Dystrophy	Recruiting	Stargardt’s Macular Dystrophy	Biological: MA09-hRPE implantation	Advanced Cell Technology	Phase 1-2	16	April 2011	December 2014
**NCT01469832**	Safety and Tolerability of Sub-retinal Transplantation of Human Embryonic Stem Cell Derived Retinal Pigmented Epithelial (hESC-RPE) Cells in Patients With Stargardt’s Macular Dystrophy (SMD)	Recruiting	Stargardt’s Macular Dystrophy|Fundus Flavimaculatus|Juvenile Macular Dystrophy	Biological: MA09-hRPE implantation	Advanced Cell Technology	Phase 1-2	16	November 2011	December 2014
**NCT01625559**	Safety and Tolerability of MA09-hRPE Cells in Patients With Stargardt’s Macular Dystrophy (SMD)	Recruiting	Stargardt’s Macular Dystrophy	Biological: MA09-hRPE implantation	CHA Bio & Diostech	Phase 1	3	September 2012	October 2014
**NCT01632527**	Study of Human Central Nervous System Stem Cells (HuCNS-SC) in Age-Related Macular Degeneration (AMD)	Recruiting	Age Related Macular Degeneration|Macular Degeneration|AMD	Drug: HuCNS-SC cells implantation	StemCells, Inc.	Phase 1-2	16	June 2012	June 2014
**NCT01674829**	A Phase I/IIa, Open-Label, Single-Center, Prospective Study to Determine the Safety and Tolerability of Sub-retinal Transplantation of Human Embryonic Stem Cell Derived Retinal Pigmented Epithelial (MA09-hRPE) Cells in Patients With Advanced Dry Age-related Macular Degeneration (AMD)	Recruiting	Dry Age Related Macular Degeneration	Biological: MA09-hRPE implantation	CHA Bio & Diostech	Phase 1-2	12	September 2012	April 2016
**NCT01691261**	A Study Of Implantation Of Human Embryonic Stem Cell Derived Retinal Pigment Epithelium In Subjects With Acute Wet Age Related Macular Degeneration And Recent Rapid Vision Decline	Not yet recruiting	Age Related Macular Degeneration	Biological: PF-05206388 implantation	Pfizer|University College, London	Phase 1	10	April 2014	July 2016
**NCT02122159**	Research With Retinal Cells Derived From Stem Cells for Myopic Macular Degeneration	Not yet recruiting	Myopic Macular Degeneration	Biological: MA09-hRPE implantation	University of California, Los Angeles|Advanced Cell Technology, Inc.	Phase 1-2	Not specified	Not specified	Not specified

The remaining study is represented by a non-randomized safety/efficacy study phase I open label trial of RPE replacement aiming at evaluating the safety and feasibility/efficacy of treating subjects with wet AMD in whom there is rapidly progressing vision loss (*clinicaltrials.gov identifier no.NCT01691261*). The study has been sponsored by Pfizer and will be performed at University College, London (UK). It will include 10 patients that will receive Pf-05206388 RPE cells immobilized on a polyester membrane as a monolayer, derived from human ESCs. The implanted membrane is approximately 6 mm × 3 mm and is intended to be life-long.

### Clinical testing of non-neural cells for CNS diseases

While in this review we have mainly discussed ongoing clinical studies based on human NSCs or neural derivatives of pluripotent stem cells, most of the currently open Phase I/II clinical trials for treatment of brain diseases are actually testing therapeutic potential of cells of non-neural origin. Mesenchymal stem cells (MSCs) derived from bone marrow (BMD-MSCs) or adipose tissue and bone marrow derived stem cells (BMDSCs) have been proposed by some scientists and many clinicians as reliable extra-neural sources of multipotential stem cells for brain repair [62, 63]. From a clinical point of view, these non-neural cell sources provide the advantage of easy accessibility and of autologous approaches, thus minimizing immune reactions. Nonetheless, although these cells have been widely used in clinical settings for non neural diseases and their safety has been demonstrated when injected in several body districts (different from the CNS), particularly with autologous transplants, their sustained therapeutic benefit for brain disorders has not been consistently obtained, neither in preclinical nor in clinical studies. Several optimistic reports have been published regarding MSCs conversion into NSCs or neurons but actually the proof of functional neurons generation from MSCs is still missing. Indeed, these reports based their conclusions on morphological inspection or on the expression of few neuronal markers rather than demonstrating that these neuronal-like cells exhibit all the morphological, antigenic and functional key properties of neurons (i.e. presence of mature synaptic structures, electrical excitability, controlled neurotransmitter release) or showing an effect in disease models [64, 65]. Overall, the currently available evidences indicate that the number of BMDSCs or MSCs able to differentiate into neurons, if any, is extremely low and irrelevant when thinking of their potential clinical exploitation. However, several laboratories continue to declare that MSCs can be converted into neurons, thus encouraging many unsubstantiated clinical trials testing these cells for brain repair. To this regard, the clinical outcome published in 2010 regarding the first open-label pilot clinical trial with autologous BMD-MSCs transplanted into the striatum of patients with advanced Parkinson’s Disease is emblematic [66]. Seven PD patients aged 22 to 62 years received a single-dose (1 million per kg body weight) unilateral transplantation of autologous cells into the sublateral ventricular zone by stereotaxic surgery. Patients were followed up for a period that ranged from 10 to 36 months and the results indicate that the protocol seems to be safe with no adverse events occurring during the observation period. Nonetheless, the number of patients recruited and the uncontrolled type of trial did not permit demonstration of effectiveness of the treatment. Indeed, the clinical improvements were only marginal and possibly due to a placebo effect. Regardless of the capability of MSCs to differentiate into neurons, many trials have been pushed by the belief that MSCs might provide benefit to patients affected from brain diseases by virtue of neuroprotective/immunomodulatory properties, thus supporting diseased cells and controlling or adjusting inflammation within the patient’s CNS. As such, many preclinical studies have been performed with MSCs but at the present time it is yet unproven that they might exert substantial and enduring effects in any neurological condition. Finally, long-term follow up should be performed in order to be confident about the safety of injecting MSCs (or any other non-neural cell type) in the brain. Indeed, it is still unknown (i) what eventually happens to these cells in the brain, (ii) if they survive long term in the lesioned region and (iii) if their “ectopic nature” might induce adverse effects in the future.

Beside the use of MSCs and BD-MSCs, hematopoietic stem cells (HSCs) represent an interesting source of non neural cells, exploitable for treating some CNS disorders. Indeed, HSCs have shown very promising clinical results for gene therapy treatments of congenital leukodystrophies [67, 68].

## Conclusions

NSCs have become one of the most intensively studied cell types in biology. Our knowledge of their identities and properties has been radically revolutionized by the possibility to isolate and expand them *in vitro*. One can anticipate that a rigorous assessment of the functional qualities of NSCs combined with emerging knowledge of CNS microenvironment following injury will allow us to exploit the advantages offered by these cells in the clinical setting. It should be emphasized that the NSCs potential to regenerate the brain relies also on the competence of other cells to contribute to repairing processes. NSCs and pluripotent stem cells neural derivatives are now under scrutiny in early Phase I/II trials for CNS disorders. Also new systems based on direct conversion of non neural cells into NSCs and into specific neuronal populations are bringing new possible strategies of intervention. While it is yet too early to predict the outcome of these trials, initial results indicate no safety concerns. Although the field is moving forward every year and new trials are continuously being planned and started, to date none was successful, thus reducing the hope that stem cells may be a valid CNS therapy in the next few years.

Before envisaging any therapeutic application of such cells for CNS disorders, we need to confront several, and still unsolved, problems: (i) the ideal stem cell source for transplantation in each specific disease context; (ii) the appropriate number of cells to transplant; (iii) a clinically-applicable transplantation strategy; (iv) the right disease stage for cell transplantation; and, finally, (v) the most appropriate *in vivo* and/or *in vitro* manipulations to obtain the proper cells to be transplanted. With time, progress will be made, but it is only by following a correct preclinical research and well-established methods of translation from the laboratory to the clinic that this can happen. The growing interest and participation in stem cell therapies of big pharmaceutical companies and their collaborating partners worldwide will represent an important step in order to increase the number of new well-defined clinical trials in the next few years. It is mandatory that scientists and clinicians should work side by side in order to pay attention not to abandon these principles in the hurry to move to clinic and to responsibly communicate to the public and patients [69]. The real risk is that the entire field of stem cell research and therapies might become a fertile ground for commercially driven illusion sellers that peddle the poisoning concept of stem cell research as an alchemic science.
